# Contribution of Drinking Water Softeners to Daily Phosphate Intake in Slovenia

**DOI:** 10.3390/ijerph14101186

**Published:** 2017-10-06

**Authors:** Gregor Jereb, Borut Poljšak, Ivan Eržen

**Affiliations:** 1 Faculty of Health Sciences, University of Ljubljana, Zdravstvena pot 5, 1000 Ljubljana, Slovenia; gregor.jereb@zf.uni-lj.si (G.J.); borut.poljsak@zf.uni-lj.si (B.P.); 2 Department of Environmental Health, National Institute of Public Health, Trubarjeva 2, 1000 Ljubljana, Slovenia

**Keywords:** drinking water, softening, polyphosphates, exposure assessment

## Abstract

The cumulative phosphate intake in a typical daily diet is high and, according to several studies, already exceeds recommended values. The exposure of the general population to phosphorus via drinking water is generally not known. One of the hidden sources of phosphorus in a daily diet is sodium polyphosphate, commonly used as a drinking water softener. In Slovenia, softening of drinking water is carried out exclusively within the internal (household) drinking water supply systems to prevent the accumulation of limescale. The aim of the study was to determine the prevalence of sodium phosphates in the drinking water in Slovenia in different types of buildings, to determine residents’ awareness of the presence of chemical softeners in their drinking water, and to provide an exposure assessment on the phosphorus intake from drinking water. In the current study, the presence of phosphates in the samples of drinking water was determined using a spectrophotometric method with ammonium molybdate. In nearly half of the samples, the presence of phosphates as water softeners was confirmed. The measured concentrations varied substantially from 0.2 mg PO_4_/L to 24.6 mg PO_4_/L. Nearly 70% of the respondents were not familiar with the exact data on water softening in their buildings. It follows that concentrations of added phosphates should be controlled and the consumers should be informed of the added chemicals in their drinking water. The health risks of using sodium polyphosphate as a drinking water softener have not been sufficiently investigated and assessed. It is highly recommended that proper guidelines and regulations are developed and introduced to protect human health from adverse effects of chemicals in water intended for human consumption.

## 1. Introduction

The intake of phosphorus through various sources in daily diet is high [1,2,3]. Estimated dietary intake of phosphates has been increasing in recent years, especially in developed countries [4,5]. This is predominantly due to the high amount of phosphate additives in pre-processed food [6,7,8,9]. According to Bussche et al. [10], the concentration of minerals (and also phosphates) in the urine of children varies among European countries, probably due to differences in diet and, consequently, differences in daily intake. High daily intake of phosphates represents a significant health threat [2,11,12,13,14,15,16], as a result of several different causes: elevated serum phosphate concentrations are related to renal calcification, increased kidney weight ratio, and chronic kidney disease (CKD) [11,17]. High phosphate concentrations are linked with a higher prevalence of cardiovascular disease, both in patients with CKD [18,19,20,21,22,23,24] and in healthy individuals [2,13,14,25]. Similarly, high phosphate concentrations have been linked to increased mortality among CKD patients [26,27,28] and among the general population [29,30]. A long-term high phosphate diet affects the bone structure [6,31] and the regulation of FGF23 and Klotho [17,28,32,33]. Furthermore, phosphate concentration is associated with oxidative stress [30] and, through Klotho and FGF23, adds to an aging-like syndrome [28,30,32].

One of the possible sources of phosphorus in a daily diet could be the addition of phosphate softeners to drinking water. The intake of phosphates through drinking water is often ignored during exposure assessment, despite the fact that the use of sodium polyphosphates in drinking water softening is fairly common [34,35,36,37,38,39,40]. It is, however, wrongly assumed that chemical softening is a cleaning process. Chemical water softening is primarily used to secure the drinking water infrastructure and prevent limescale formation in heat exchangers, hot-water boilers, kettles, taps, and pipes, especially when the drinking water is rather hard (carbonate hardness). It is also used to eliminate plaque which is a nuisance for the user. The main reason for drinking water softening is therefore technical, and the health effects of such treatment are underestimated or even ignored. Nonetheless, sodium polyphosphates are still commonly used as softeners, along with sodium and potassium salts in domestic drinking water distribution networks.

According to Slovenian legislation [41], using phosphates as water softeners in drinking water is not allowed, but the usage of phosphates is not regularly monitored and controlled. The exact regulations on the use of softeners have not yet been fully defined, leading to a lack of controls. Thus, further information regarding chemical softening is crucial. Softening is used rather extensively, in large residential buildings (as well as in public buildings such as schools, kindergartens and hospitals), and is predominantly administered by caretakers. The consumers are usually not aware of the chemical treatment of drinking water in their buildings and the application of phosphates is not under their control. The concentrations of phosphates may vary considerably—depending on the water softening process in individual buildings. Despite the common use of phosphates as a water softening agent, legislation in Slovenia [41] and the European Drinking Water Directive [42] do not limit phosphate levels. In fact, phosphates are not mentioned at all in these legislations. However, in the directive it states that when “preparation or distribution of water intended for human consumption may involve the use of certain substances or materials, rules are required to govern the use thereof in order to avoid possible harmful effects on human health” [42]. WHO Guidelines for drinking-water quality [43] mention the use of phosphates as corrosion inhibitors in drinking water treatment but set no limitation to its use. On the other hand, the European standard SIST EN 1212:2005 “Chemicals used for treatment of water intended for human consumption—Sodium polyphosphate” defines sodium polyphosphates as chemicals that can be used for drinking water treatment in the case of corrosion and limescale inhibition, and proposes that the treatment dose should not exceed 5 mg P_2_O_5_/L [44].

The purpose of the current study was to fill the gap regarding the information on the presence and concentrations of phosphate in drinking water. The study aimed to determine the prevalence of sodium phosphate usage in chemical drinking water softening in Slovenia. Additionally, the prevalence of sodium phosphate in the drinking water according to the type of building was determined, the residents’ awareness of the presence of chemical softeners in their drinking water was assessed, and an exposure assessment of phosphorus intake from drinking water was conducted.

## 2. Materials and Methods

### 2.1. Sample Collection

A total of 242 samples of drinking water were collected in randomly chosen blocks of flats and houses in several Slovenian cities. The study also included a short questionnaire that was distributed among the residents of houses and condos and completed on site. Predominantly, only hot water is softened (before the water enters the water heater), therefore only samples of hot water were taken for chemical analysis. Prior to sampling, the faucets were opened and flushed thoroughly for approximately 2 to 3 min until the water temperature stabilized. Water temperature was measured using a simple alcohol thermometer. After temperature stabilization, the samples were poured into chemically cleaned plastic containers with a volume of 250 to 500 mL. In the case that the chemical analyses were not carried out within a few days after sampling, the samples were deep frozen at −18 °C.

### 2.2. Chemical Analysis

Measurement of the phosphate concentration in the drinking water samples was conducted according to SIST EN ISO 6878:2004 “Water quality—Determination of phosphorus—Ammonium molybdate spectrometric method”. Solutions of ammonium molybdate and potassium antimony tartrate in an acidic medium were reacted with a diluted solution of phosphate. An intensive molybdenum blue complex formed in the presence of ascorbic acid. The color intensity of the complex is directly correlated to the phosphate concentration in the sample [45]. The standard method was modified in such a way that the analysis could be carried out in 25 mL volumetric flask. To a sample of 20 mL, 260 µL of sulphuric acid was added. Hydrolysis of polyphosphates was performed in a laboratory vacuum dryer (Kambič VS–50 SC, Semič, Slovenia), at 110 °C for 30 min. After acidic hydrolysis, the samples were cooled down and the pH was adjusted by adding 2.26 mL of sodium hydroxide, 0.5 mL of ascorbic acid, and 1 mL of reagent II (acid molybdate). The samples that contained phosphates were colored blue. The absorbance was measured at a wavelength of 880 nm with a Macherey–Nagel Nanocolor VIS (visible) spectrophotometer (Düren, Germany). The calibration curve was prepared according to the standard (SIST EN ISO 6878:2004). The curve was plotted using measurements of a series of phosphorus reference solutions (concentrations were as follows: 0.1 mg/L; 0.25 mg/L; 0.5 mg/L; 0.75 mg/L; 1.0 mg/L; 1.25 mg/L; 1.5 mg/L; 1.75 mg/L). For higher concentrations, dilution of the test portion was conducted. The calculated concentrations were expressed as a concentration of P (mg P/L) and PO_4_ (mg PO_4_/L), respectively. Each sample was analyzed in three parallels and each measurement was repeated three times. The displayed results are the average value of the aforementioned 3 × 3 repetitions. As a positive control, two standard solutions (0.5 mg P/L and 1 mg P/L) were prepared and analyzed for each batch of samples. Since the expected concentrations of phosphates were high, a 10-mm optical cell was used and the limit of detection was set at 0.2 mg PO_4_/L (0.065 mg P/L).

### 2.3. Questionnaire

During sampling, a short questionnaire was completed on site by both the house and condo residents. The questionnaire included questions about the residents’ usual water consumption and their knowledge about water softening. The question “Do you think that the water in your building is chemically softened?” was used to compare people’s awareness (knowledge) of the chemical treatment of their drinking water with the results of the presence of phosphates in the water samples. To avoid the respondents guessing, an option “don’t know” [46] was available in addition to the answers yes/no.

### 2.4. Statistical Analysis

The concentration of phosphates in hot drinking water at individual sampling sites was analyzed using descriptive statistics. A measure above or below 0.2 mg PO_4_/L (indicating the presence or no presence of added phosphates in drinking water, respectively) was used as a cut point to obtain 0/1 values. This data was then analyzed in relation to both the participants’ reported familiarity with the chemical treatment of drinking water in their buildings, and to the type of buildings (houses vs. flats/condos). A chi-square test was used to determine whether the differences in concentrations were statistically significant in relation to aforementioned independent variables. Additionally, the type of building was compared with the results of the phosphate concentrations. A chi-square test was applied that containing a 3 × 2 table (type of building vs. concentrations above/below 0.2 mg PO_4_/L).

## 3. Results

**Figure 1:**
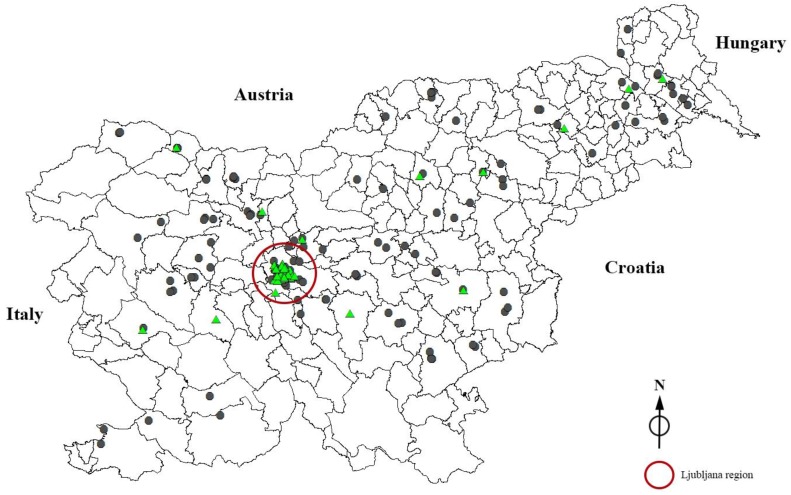
The location of sampling sites in Slovenia (● concentration of PO_4_ below the limit of detection; 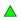 concentration of PO_4_ > 0.2 mg PO_4_/L).

The locations of the 242 samples collected in this study are presented in Figure 1. Samples were taken mainly in large residential buildings, blocks of flats, and some public buildings.

**Figure 2:**
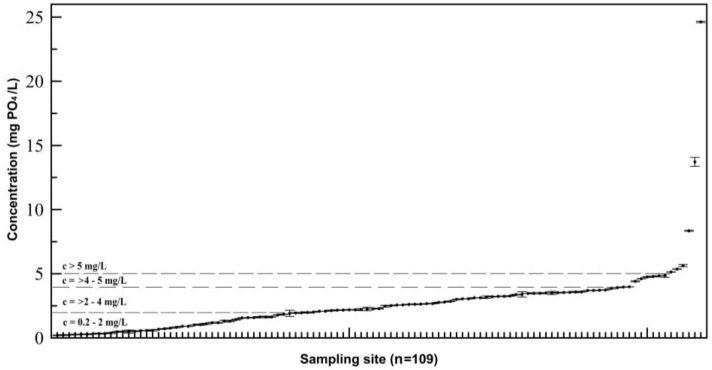
Concentrations of phosphates in mg PO_4_/L (from min. to max. concentration).

Phosphates were present in 109 out of 242 samples (45%). The concentration of individual samples varied substantially (Figure 2), ranging from 0.20 mg PO_4_/L (0.07 mg P/L) to 24.62 mg PO_4_/L (8.03 mg P/L). The measured concentrations exceeded even the recommended treatment dose of the European standard [44] several times in three samples. Most of the positive samples (c > 0.2 mg PO_4_/L) were recorded in Ljubljana (89%) and the remaining (11%) positive samples were found in various locations across Slovenia. There is an inconsistency in the chemical softening process due to the rather high fluctuation of phosphate concentrations. This fluctuation clearly indicates the application of inexact quantities and poor control over administering chemicals in drinking water.

The measured concentrations in 43 samples ranged from 0.2 to 2 mg PO_4_/L, in 54 samples the concentrations were between 2 and 4 mg PO_4_/L, and in 12 samples the concentration exceeded 4 mg PO_4_/L, while in 6 samples it was higher than 5 mg PO_4_/L.

Statistically significant differences were established in the distribution of the measured concentrations (above or below 0.2 mg PO_4_/L) in relation to the identified type of the buildings (χ^2^ = 24.315, df = 2, *p* < 0.001). In houses, as many as 92% (*n* = 33) of all samples were negative (no presence of phosphates in drinking water samples was found) and only 8% (*n* = 3) of the samples contained phosphates. In blocks of flats as well as in public buildings (kindergartens, schools, and student dorms) phosphates were commonly used as a hot drinking water softener: specifically, in 47% (*n* = 51) of blocks of flats and 55% (*n* = 55) of public buildings.

In each of the buildings where the samples were taken one of the dwellers answered a question about whether they knew if drinking water in their building was chemically softened. Table 1 shows the answers to the following question: “Is water in your building chemically treated or not?”. A statistically significant difference was found in the distribution of the respondents’ answers in regards to the measured concentrations of phosphates (values above or below 0.2 mg PO_4_/L were treated as 1 and 0, respectively) in their drinking water (χ^2^ = 22.616, df = 2, *p* < 0.001). The results indicate that 37% of respondents thought that the drinking water in their building was not chemically softened. A vast majority of respondents were not familiar with the chemical treatment of drinking water in their buildings. Almost half of the respondents (49%) did not know if their drinking water was chemically treated. Among those who claimed that they know the answer (“yes” or “no” for chemical treatment) only 30% answered correctly. However, for 20% of the respondents, the answer was inversely proportional to the actual concentration. It can be concluded that the vast majority (70%) of users do not know whether drinking water softeners (polyphosphates) are used in their buildings.

The contribution to the total RDA (Recommended Dietary Allowance) of phosphorus through drinking water consumption was calculated (Table 2). Three different scenarios were considered: (i) a “worst case” scenario with the assumption that all consumed water is softened with the highest concentration measured in our study (8.03 mg P/L or 24.6 mgPO_4_/L); (ii) a “high concentration” scenario with the assumption that all consumed water is burdened with the 95 percentile concentration (1.71 mg P/L or 5.2 mgPO_4_/L); and (iii) a “realistic” scenario where the median concentration (0.75 mg P/L or 2.3 mgPO_4_/L) from the presented study was taken into account. The consumption of water was calculated according to age: 2 L (for nine years and above), 1 L (for one to eight years), and 0.75 L (for infants under one year) per day [47,48]. According to the RDA [49], drinking water consumption could contribute to a daily phosphorus intake of 0.2 and 0.1% in children and adults, respectively (in the case of the realistic scenario), and up to 0.5 percent in both children and adults in the case of a high phosphate concentration in the drinking water. Furthermore, an extremely high phosphate concentration in drinking water (as measured in the current study) can contribute to even more than 2% of the daily requirement of phosphorus. In the case of infants, intake in the realistic scenario contributes 0.6% of the Adequate Intake (AI), while intake in the worst-case scenario could contribute up to 6% of infant AI, solely from drinking water.

## 4. Discussion

Since the identified dietary intake of phosphorus dramatically exceeds [1,2,3] the recommended values [49], it is necessary to consider all routes of administration in the management plan in order to reduce the intake of phosphorus.

Sodium polyphosphates are commonly used in Slovenia for the prevention of limescale formation, reducing the costs of maintenance and reconstruction of water supply networks. However, the potential health risks are currently not taken into consideration. The use of chemicals for drinking water softening is not subject to health inspection and there is no monitoring of their presence in drinking water. The concentrations of drinking water softeners are not under proper control and therefore vary greatly.

The results of this study clearly indicate that sodium polyphosphates as a drinking water softener are rather commonly used in Slovenia. In 45% of the collected samples of hot drinking water across different parts of the country the presence of phosphates was confirmed. The majority of samples were collected in Ljubljana, the capital of Slovenia, where water is supplied from the same aquifer. It has, therefore, similar physiochemical characteristics and consequently the same carbonate hardness of 150 mg/L CaO (2.6749 mmol CaCO_3_/L). Despite these common characteristics, the concentrations of phosphates in drinking water vary significantly, from 0.2 mg PO_4_/L to 24.6 mg PO_4_/L, a factor of more than 100. This clearly indicates an incoherent practice by individual caretakers in different blocks of flats and points to the need for proper guidelines and control.

A comparison of the concentrations of phosphates measured in this study with literature was not possible since no similar published studies were found in Scopus / WOS / PubMed, using the key words: “phosphate AND concentration AND drinking AND water”. Although prior studies explored the correlation between phosphate concentrations (defining 0.6–1.5 mg P/L as a usual phosphate concentration range) and water pipes corrosion inhibition [36,37,38,40,50,51,52] and other studies explored biofilm responses to phosphate load [53,54,55,56], no systematic study of phosphate concentration in drinking water systems is available. McNeill and Edwards [37] conducted a survey on US water utilities which concluded that the phosphate dosage in drinking water ranges from 0.2 to 3 mg PO_4_/L. However, a limitation of their study is that the concentrations were not measured but collected only as reported dosages of phosphates added, so data on the actual concentration are not known. In approximately 55% of drinking water utilities, phosphate water treatment was reported. These results are generally in accordance with those of the present study. However, in this study concentrations even higher than 3 mg PO_4_/L were found in 38% of cases. Furthermore, in Slovenia water is not softened in a drinking water facility, water softening is performed only in the heat stations of households.

Since, in Slovenia, no relevant data regarding drinking water softening and phosphate concentration in drinking water are available, sampling was carried out across the entire country. The majority of samples were collected in the capital, mostly in large blocks of flats with a high number of individuals exposed to water softening. In contrast to the residents of separate houses, the residents of larger buildings were usually not aware of the chemical treatment of their water and did not have any information regarding water softening. As expected, chemical softening is predominantly used in large blocks of flats as well as in public buildings (kindergartens, schools, student dorms, and hospitals) and rarely used in separate houses. In all probability, the caretakers of larger buildings use chemical softeners in order to prevent limescale formation and facilitate the maintenance of the water pipe system. It is disturbing that the majority of inhabitants surveyed have no information regarding phosphate additives. A major part of the inhabitants did not know whether their drinking water is chemically treated or not. Besides the 49.2% of inhabitants who reported that they did not know anything about water softening, 20.7% of respondents’ answers were not correct. It can be concluded that more than half (nearly 70%) of respondents were not familiar with the exact data on water softening. Only 30.1% of the answers were consistent with the results of the chemical analyses of water samples (4.1% stated that their water was softened and 26% that it was not).

The intake of phosphorus via individual food products is usually not extremely high and could even be negligible with respect to the RDA. However, the daily intake of phosphorus due to accumulation is of great importance [4,5]. In the case of phosphate-based drinking water softeners, the recommended values of phosphates are much lower than the RDA, but drinking water consumption still contributes to the total amount of daily intake. The intake via drinking water in the worst-case scenario (all consumed water is chemically treated with the highest concentration of phosphates) represents between 1.3% and 2.3% of the RDA, and, in the case of infants it is as high as 6% of the AI. In the case of the realistic scenario (using the median measured concentration in the present study) the contribution to the RDA ranges from 0.1 to 0.2%.

It is necessary to emphasize that the exposure to phosphorus via drinking water is generally not anticipated. According to Andjelov et al. [57] the concentration of phosphorus in the ground water in Ljubljansko Polje (the aquifer for the Ljubljana region, where the majority of positive samples were confirmed) ranges between 26 and 60 µg/L, depending on the location of ground water sampling. This concentration is 3.3 to 7.6 times lower that detection limit in our investigation.

Furthermore, since the intake of phosphorus through a typical daily diet is too high [1,2,3] and already exceeds daily requirements, no additional phosphate intake, which might be a health risk for the consumer, is needed. It should be pointed out that the calculated values of exposure were based on the assumption that all consumed water was hot water.

Based on the latest study findings on health effects of high phosphorus intake, the EFSA (European Food Safety Authority) undertake a re-evaluation of phosphates for use as food additives with a high priority by 31 December 2018 [12]. Exposure assessment of phosphorus daily intake should also include the amount of phosphates consumed by drinking water.

Furthermore, consumers should be informed about the additives and added chemicals in their food, including drinking water. It follows that users should be properly informed about the chemical treatment and the concentrations of phosphates in their drinking water. In addition, the sodium daily intake should also be considered for sodium polyphosphate risk assessment, especially in the case of the more vulnerable groups in the population (people suffering from phosphatemia or acute phosphate nephropathy, etc.).

The study produced results which indicate that the health risks of sodium polyphosphate as a drinking water softener are not sufficiently investigated and assessed. Consequently, the health risks of exceeded phosphate values may be underestimated. Further studies on the current topic are therefore recommended. It is necessary to develop guidelines and adopt regulations on the use of phosphates in drinking water. Centralized (professional) water softening without sodium polyphosphates could be one of the solutions.

## 5. Conclusions

Several conclusions can be drawn from the study presented. The first major finding was that in 45% of the samples taken at different locations across Slovenia, the presence of phosphates as a water softener was confirmed. The measured concentrations vary substantially from 0.2 mg PO_4_/L to 24.6 mg PO_4_/L. Furthermore, more than half (70%) of the survey participants were not familiar with the exact data on water softening. The results of this research indicate that the health risks of sodium polyphosphate as a drinking water softener are not sufficiently investigated and addressed. From a public health perspective, it is important that risk reduction measures be implemented to reduce the total intake of phosphate. It is necessary that proper guidelines, regulations, and control regarding drinking water softening and phosphate concentration are adopted and applied.

## Figures and Tables

**Table 1:** Respondents’ answers regarding chemical treatment and measured phosphate concentrations in drinking water.

Measured Concentration (mg PO_4_/L) *	Respondents’ Answers Regarding Chemical Softening of Their Drinking Water		
	Yes % (*n*)	No % (*n*)	Do not Know % (*n*)
c < 0.2	9.5% (23)	26.0% (63)	19.4% (47)
c > 0.2	4.1% (10)	11.2% (27)	29.8% (72)
Total	13.6% (33)	37.2% (90)	49.2% (119)

* the measured concentration of phosphates; above and below 0.2 mg PO_4_/L in regards to the detection limit of the measurement procedure.

**Table 2:** Exposure assessment for phosphorus intake by drinking water.

Age (years)	RDA * (mg/day)	Contribution ** (%) of Phosphates in Drinking Water to RDA		
		Worst Case Scenario ***	High Concentration Scenario ****	Realistic Scenario *****
0–6 months	100 (AI)	6.0	1.3	0.6
6–12 months	275 (AI)	2.2	0.5	0.2
1–3	460	1.7	0.4	0.2
4–8	500	1.6	0.3	0.2
9–13	1250	1.3	0.3	0.1
14–18	1250	1.3	0.3	0.1
19–30	700	2.3	0.5	0.2
31–50	700	2.3	0.5	0.2
51–70	700	2.3	0.5	0.2
>71	700	2.3	0.5	0.2

* recommended dietary allowance [49], in the case of infants AI—Adequate Intake [49]; ** daily water consumption = 2 L for 9 years and above, 1 L for 1 to 8 years, and 0.75 L for infants under 1 year [47,48]; *** highest measured concentration = 8 mg P/L; **** 95 percentiles of measured concentration = 1.71 mg P/L; ***** median value of measured concentration = 0.75 mg P/L.
